# Phenology, growth, yield, and yield-related traits of Ethiopian garlic genotypes. A review

**DOI:** 10.1016/j.heliyon.2023.e16497

**Published:** 2023-05-22

**Authors:** Gebre Garmame Galgaye

**Affiliations:** Department of Horticulture, Bule Hora University, Ethiopia

**Keywords:** Ethiopia, Garlic yield, Genetic variability, Growth traits, Yield parameters

## Abstract

Garlic is one of the most important medicinal plants and consists of high sulfur concentration compounds like diallyl disulfide, allicin, diallyl trisulfide, and *S*-allylcysteine. It contributes for anti-inflammatory, anticancer, anthelmintics, antifungal, liver protection, antioxidants, antistress, and wound healing properties. Therefore, garlic plays a significant role in human life. To popularize this multi-purpose crop in Ethiopia, there are ample opportunities and potential, like a wide agro-ecology and a labor force. However, today's garlic production in Ethiopia is low because of different factors like climate, soil, microbiome and cultural practices. While, mainly due to genotype variation, garlic yield is constrained. Thus, gathering and reviewing the impact of genotypes on phenology, growth, yield, and yield traits is important to summarize and organize. Hence, the main objective of this paper is to show the phenology, growth, yield, and yield-related traits of garlic as influenced by genotype. Finally, this review scoped to reveal phenology, growth, yield and yield traits as influenced by Ethiopian garlic genotypes. Generally, different genotypes influence the phenology, growth, yield, and yield traits of garlic. Therefore, garlic producers should use a high-yielding variety (genotype) in the area.

## 1 Introduction

Garlic (*Allium sativum* L.) belongs to the family Alliaceous, genus Allium, and originated in Russia, West China, Afghanistan, and India [1]. It was spread to other parts of the world through trade and colonization [2,3]. It has been food and medicine for a long time in India and China for more than 5000 years, while in Egypt before 2000 B.C [1]. Today, China is the largest producer and consumer of garlic in the world [2]. Garlic is one of the most expensive cash crops for Ethiopia's low-income smallholder producers. In 2019/20 and 2020/21, a high net return of USD 19,963.2 and USD 17,263.2 per hectare was reported, respectively [4]. Consequently, farmers produce garlic for home consumption and also as an income source [5]. Besides, Azene [6] reported that, due to the high sulfur concentration of compounds like diallyl disulfide, allicin, diallyl trisulfide, and *S*-allylcysteine in garlic. It has anti-inflammatory, therapeutic, ulcer-inhibiting, antidiabetic, rheumatologic antimicrobial, analgesic, anticholinergic, anticancer, anthelmintics, antifungal, liver protection, antioxidants, antistress, and wound healing properties [7,8]. Not only this, but also several scholars indicate that garlic has properties that could be used to treat diseases including arthritis, asthma, tuberculosis, malaria, chronic fever, leprosy, runny nose, skin discoloration, itching, tumors, indigestion, an enlarged spleen, colic, heart problems, fistula, hemorrhoids, gout, bone fracture, urinary tract, worms, kidney stones, jaundice, headache, cataract, night blindness, and epilepsy [[6], [7], [8], [9], [10]]. Therefore, garlic plays a significant role in human life. To popularize this multi-purpose crop in Ethiopia, there are ample opportunities and potential, like a wide agro-ecology and a labor force. However, today's garlic production in Ethiopia is low [[11], [12], [13], [14], [15], [16]]. The total production from 12,429 ha of land was only 124,801 quartiles. The low production of garlic growth, yield, and yield-related traits was due to biotic and abiotic factors such as lack of high-yielding genotypes [[16], [17], [18], [19], [20], [21], [22], [23], [24], [25], [26], [27], [28], [29], [30], [31], [32], [33], [34], [35]], inappropriate organic and inorganic fertilizer [3,4,18,[36], [37], [38], [39], [40], [41], [42], [43], [44], [45], [46], [47], [48], [49], [50], [51], [52]], inappropriate intra-row spacing [38,42,43,[53], [54], [55], [56],^69^], clove weight and size [[57], [58], [59], [60]], watering [[61], [62], [63], [64], [65], [66], [67], [68]], mulching [70,71], disease [12,36,42,[53], [54], [55],[72], [73], [74], [75], [76], [77], [78], [79], [80], [81], [82], [83], [84], [85], [86], [87], [88]], postharvest [[89], [90], [91], [92], [93]], and climate change [103].

Among them, the most important primary factor is genotype due to its genetic variability at different locations (agronomy, post-harvest, and institutional) [[16], [17], [18], [19], [20], [21], [22], [23], [24], [25], [26], [27], [28], [29], [30], [31], [32], [33], [34], [35],[89], [90], [91], [92], [93], [94], [95], [96], [97], [98], [99], [100], [101], [102],[104], [105], [106], [107], [108]]. This review focuses on impact of genotypes on phenology, growth, yield and yield traits in Ethiopia only. To fill the information gap regarding to impact of genotypes on phenology, growth, yield and yield traits in Ethiopia Garlic is grown in all temperate and subtropical regions as well as in hilly lowland areas all over the world [105]. While in Ethiopia, it is commonly produced in the midland and highlands [[26], [27], [28], [29],^31^,^107^,^108^]. Scholars have shown that the genotype of the garlic plant affects the growth yield and yield-related traits of the garlic plant [17,24,26,27]. Mohammed et al. [17] reported that among the types of varieties like Chefe, Kuriftu, Tsedey 92, Holleta, Chelenko I (Standard Check), and Local, there were significant effects on the plant height, leaf length, bulb diameter, number of cloves per bulb, bulb weight, and yield. Besides, other genotypes exist, such as Briki-Gc-1/16, Birki-Gc-2/16, Bora-Gc-16, Bisheftu Nech, Bora-2/16, Bora-3/16, Bora-1/16, MM-98, Sekela-Lijima, Dejen-Koncher, Awebel-Dehguma, Dejen-Borbor, Bure-Kebsa, Sinan-Debre Zeit, Sekela-Yedem Mariyam, Banja-Satma, Dejen-Gibgib, Sekela-Menbeta, Banja-Satma, Awebel-Yazera Giorgis, Dembecha-Senseb Gebriel, Dejen-Jeva, G-99-2, Felegdaero, Guahgot, and other locals were tested. Consequently, genotypes influenced growth, yield, and yield-related traits in different areas of the country (Ethiopia) [18,19,21,22,24,26,27,[30], [31], [32], [33], [34], [35]]. Gebre [114] indicated that garlic yield was determined by different factors in Ethiopia. Besides, the influence of garlic yield varies from place to place. However, in Ethiopia, no one has reviewed and summarized the influence of garlic genotype on phenology, growth, yield, and yield traits. Therefore, countrywide (Ethiopia), gathering and reviewing the impact of genotypes on phenology, growth, yield, and yield traits is important to summarize and organize as insight for researchers, students, investors, and businessmen. Hence, the main objective of this review is to show the growth, yield, and yield-related traits of garlic as influenced by genotype in Ethiopia.

## 2 Literature review

### 2.1 Garlic origin, distribution, and importance

*Allium sativum* L. (2n = 16) is one of the plants belonging to the genus Allium (Alliaceous), which is one of the largest monocot genera, consisting of more than 900 species distributed in the northern hemisphere [112,113].

Most scientists agree that garlic has been used as a medicinal plant and a food source for a long time (over 7000 years) [[6], [7], [8], [9], [10]]. In line with this brief history, Kamenetsky [111] indicates that garlic originated on the northwestern side of the Tien-Shan Mountains of Kirgizia in central Asia (Kazakhstan). There is also evidence that it has been in use for a long time in Egypt before 2000 B.C., and also in India and China for more than 5000 years. China is the largest producer and consumer of garlic in the world today [57,112]. Garlic is closely related to shallots, onions, rakkyo, chives, and leeks [110]. As previously stated, garlic has been used for medicinal purposes since the dawn of time. It was reported that many ancient people used garlic for medical purposes to boost strength and hard work. Garlic, whether in its raw or processed form to different end products such as oil, powder form, and water extracts, played an important and crucial role in the beginning of science (chemistry and modern medicine). Garlic compounds such as diallyl thiosulfinate, allicin, diallyl mono-, di-, and trisulfides, and alliin treat various diseases [[6], [7], [8], [9], [10],[110], [111], [112],^112^,^113^].

### 2.2 Production and productivity of garlic in Ethiopia

Garlic has been cultivated in different parts of the country, mostly in Oromia, Amahara, Tigray, and southern Ethiopia [114]. This might be due to the fact that the most suitable production opportunity areas of the country are in Oromia, Amahara, Tigray, and Southern Ethiopia. This area is blessed by broad potentials such as a wide agro-ecology consisting of water resources, bimodal rainfall distribution, soil fertility, and country-wide policies and initiatives. In Ethiopia, well-known improved and cultivated varieties include Kuriftu, Holleta, Bishoftu Nechi, Tseday 92, Chelenko I, and Chafe. Aside from that, various local cultivars were grown in the backyards of Ethiopian farmers [114]. However, the production system for garlic in Ethiopia is still traditional and not modernized. In 2019/20 and 2020/21, garlic production coverage in Ethiopia was 18,344.46 ha and 15,979.54 ha, respectively, which is reduced by 12.89% [4]. As a result, garlic yields in 2019–2020 and 2020–2021 were reduced by 13.52%–83.18 qt/ha and 71.93 qt/ha, respectively. Gebre [114] revealed that the reduction of garlic production and productivity in Ethiopia was affected by different institutional, farmer, agronomic, and post-harvest determinants [91,95]. The main challenges confronting Ethiopian farmers are institutional factors such as insufficient credit service, a lack of high yielding and pest resistant varieties, a lack of improved seed providing services, the supply of chemicals and irrigation facilities, a lack of market linkage and information, and a lack of appropriate farmer level extension. Farmers characterized by illiteracy, less experience in garlic production, limited skill sets, and a negative attitude towards agricultural innovations and technologies could also hinder garlic production and productivity [104]. In addition, inappropriate use of agronomic practices like selection of variety at a specific area based on variety character, plant population per area, mulching, watering, fertilization, and harvesting stage applied by farmers was detrimental to garlic production and productivity in Ethiopia [91,95,97]. Furthermore, due to a lack of good post-harvest practices like transportation facilities, storage conditions, marketing, and processing issues, Ethiopian farmers were challenged for a long period of time, even today [97].

### 2.3 Influence of garlic genotype on phenology, growth, yield, and yield traits in Ethiopia

Several researchers had reported that genetic diversity in garlic is revealed in genotypes that are tested for a variety of different morphological (growth, yield, and yield traits) traits that indicate the presence of true variability in the garlic [[17], [18], [19], [20], [21], [22], [23], [24], [25], [26], [27], [28], [29], [30], [31], [32], [33]]. Azene [56] indicated that there was great variability among the garlic accessions for leaf length (cm), number of leaves per plant (cm), bulb weight (g), bulb diameter (equatorial) (cm), number of cloves per bulb, clove weight (g), and bulb yield (tons per hectare). Moreover, different studies agree with the study mentioned above [56], which revealed significant variability in Ethiopian garlic genotypes for phonological traits like days to 50% emergence and days to 75% physiological maturity, growth traits such as pseudo plant height, leaf width, leaf area, leaf area index, and yield traits like clove length and clove diameter, biomass, harvest index, and marketable and nonmarketable yield [18,19,21,27,31,35]. Therefore, each trait is discussed as follows.

#### 2.3.1 Influence of genotypes on the phenology of garlic in Ethiopia

##### 2.3.1.1 Influence of garlic genotype on days to 50% emergence in Ethiopia

Numerous scientific papers indicate that the phenology of garlic (days to 50% plant emergence and days to 75% physiological maturity) was found to be significantly varied by genotypes (18–19, 24–30, and 31–32).

According to Tadesse [18], a higher number of days to emerge was observed in an improved variety called Tsedey (15.55 days after planting), whereas the Guahgot local cultivar (10.5 days) has emerged earlier. Similarly, Tesfaye et al. [31] discovered that, among the total of 49 garlic accessions collected from east and west Arsi, Arsi, north Shewa, Sidama (region), and Bale zones of Ethiopia, days to emergence were highly affected by garlic accessions. This could indicates the presence of real genetic variability for use in future garlic varietal breeding. The former variety was achieved at 50% emergence within 10 DAP (Days after Planting) [19] indicating the presence of real genetic variability for use in future garlic varietal breeding. Furthermore, Ayalew et al. [19] discovered that variety has a highly significant impact on days to 50% emergence, with Bishoftu Nech and MM-98 varieties being early and late, respectively. The former variety was achieved at 50% emergence within 10 DAP (Days after Planting) [19]. At 12 DAP, local varieties [1] and Kuriftu achieved 50% emergence [19].

##### 2.3.1.2 Influence of genotypes on days to 75% physiological maturity of garlic in Ethiopia

Maturity in plant physiology is the stage of capability for further development or ripening that is ready for harvesting, eating, and processing. Therefore, this stage is critical for garlic, which could be affected by different genotypes. Therefore, this stage is critical for garlic, which could be affected by different genotypes. Consequently, real differences were reported by various scholars [18,19,24]. The Guahgot local variety matured 19 days later (158.67 days) than the improved cultivars Felegdaero and Kuriftu [18]. In line with Tadesse's [18] investigation, a study conducted during dry periods under irrigation in Northwestern Ethiopia reported that the Bishoftu Nech variety matured (135 DAP), followed by Kuriftu (143 DAP), while MM-98 matured late at 176 DAP [19]. According to Mulu et al. [24], days to maturity range from 127.4 to 140.4 days, indicating that Tsedey 92 has taken a long time (140.4 DAP) to mature whereas the local variety has taken a short time (127.4) to mature. Contrarily, Tsedey 92 has matured earlier (at 126 DAP) than the local cultivar, which has taken more than seven days to reach maturity. Supporting this, Tadese [30] indicated that a local variety was found to be a relatively late-maturing variety (140 days) as compared to others considered in the experiment. G-99-2 was recorded as a relatively early maturing variety (127 DAP), which is similar to G-161-2 (129 DAP), followed by Bisheftu Netch (131 DAP) and Tsedey 92 (13). Non-significant differences were observed among varieties such as Holeta (138.6 days), Chafe (139.6), Tsedey 92 (140.4 DAP), and local for 50% of the plants to emerge [17]. On the other hand, cultivar Chefe was recorded as an early maturing cultivar that has taken only 95 DAP and 94 DAP at Angot and Woreta in northern Ethiopia, respectively [26]. Similarly, earlier accession indications indicated that the accessions only required 97 to 130 DAP to mature. An accession, G19/03 (97 DAP), was recorded, and the late-maturing accessions, namely (122.5–131 DAP) G-28/03, G-30/03, G-31/03, G-32/03, G-33/03, G-37/03, G-59/03, G-69/03, G-74/03, and G-77/03, were revealed [31]. Different germplasm collected from various southern Ethiopian areas found to be highly different in terms of days to maturity. The first germplasm was G5 (108 DAP), which was followed by G18 from Awebel-Yazera giorgis (109.33 DAP), G13 from Dejen-Gibgib (109.33 DAP), G5 from Dembecha-Senseb Gebriel (109 DAP), G38 from G48 from Sekela-Yedem Mariyam, and G50 from Sekela-Menbeta [27]. Other studies have found that accession GOG-008/18 matured quickly (116.50 DAP), followed by GOG-006/18 (117.00 DAP), and GOG-006/18 (117.25 DAP), whereas accession GOG-072/18 and GOG-079/18 matured slowly (135.25 DAP), followed by GOG-065/18 (135.00 DAP) [21].

#### 2.3.2 Influence of genotypes on garlic growth traits in Ethiopia

##### 2.3.2.1 Influence of genotype on pseudo plant height of garlic in Ethiopia

Moles et al. [109] stated that pseudo-plant height refers to the central part of the plant's ecological strategy, which is strongly and positively correlated with life span, yield mass, and time to 75% maturity. Therefore, the garlic pseudo-plant height in plant growth is too crucial. Several researchers reported that garlic pseudo-plant height could be significantly influenced by garlic variety. Ayalew [19] revealed that the pseudo plant height of garlic was significantly affected by garlic varieties (p 0.01), in which the significantly highest pseudo plant height was observed from the local variety (28.80 cm), followed by Kuriftu (24.53 cm), and MM-98 (24.43 cm), while the shortest pseudo plant height was recorded from Bishoftu-Nech (22.31 cm), respectively. On the other hand, Mohammed et al. [17] indicated that the tallest pseudo plant height (55.29 cm) was recorded from the Chelenko I variety, followed by the local (51.52 cm) and Tsedey (48.98 cm) varieties, whereas the shortest pseudo-plant height (42.37 cm) of garlic was observed from Holata [17]. However, a contrary report was revealed that Holleta (57.43 cm) found to be the tallest variety in the Angot area, northern Ethiopia [26]. Besides, varieties Tsedey 92 and local varieties found to be the tallest (68.26 cm) and shortest (62.02 cm) in pseudo-plant height, respectively [24]. Tadese [30] reported that the highest significant difference in pseudo-plant height (55.94 cm) was also observed in variety Tsedey 92, while the lowest (45.22 cm) is from the local variety. However, it was not indicated a real difference in garlic pseudo plant height among the improved varieties and cultivars considered in the experiment [26,30]. Whereas Adiszemene local (43.63 cm) was found to be shorter in pseudo plant height than other cultivars tested in the Woreta area, Chefe (42.65 cm) and Adiszemene local (41.60 cm) were found to be the shortest cultivars in the Ginza area. Abreham et al. [34] clearly indicated that the pseudo-plant height of the Tsedey 92 variety was recorded as 2.23, 1.62, and 2.32 cm taller than the local variety at 50, 70, and 90 days after emergence, respectively. Indeed, a wide variation was observed in pseudo-plant height among the accessions, indicating that genotypes G-50/03 and G-7/2003 had the shortest pseudo-plant height (43.65 cm) [31]. Furthermore, a study conducted by Atinafu et al. [21] on 55 garlic germplasm samples, three standard checks, and three promising genotypes revealed that garlic accessions significantly differed in terms of pseudo-plant height, with GOG-065/18 being the tallest (64.90 cm), followed by GOG-072/18 (64.65 cm), GOG-069/18 (62.90 cm), and genotype GOG-018 (44.25 cm). It is not surprising that very high significant (p 0.0001) differences among germplasm have elucidated that the tallest pseudo-plant height of 72.83 cm was observed in germplasm collected from the Dembecha-Senseb Gebriel area, which is statistically similar to germplasms G13 and G11 collected from Dejen-Gibgib (66.78 cm) and Dejen-Jeva (66.22 cm), respectively. The shortest plant height (43.44 cm) was recorded from germplasm G24 collected from Sekela-Yedem Mariyam [27]. For the garlic pseudo-plant height studied, the variations reviewed in different scientific studies revealed the existence of genetic variation among varieties, genotypes, cultivars, accessions, and germplasms that interacted with different areas of the country (Ethiopia), and there is a modest but significant potential for a future Ethiopian garlic improvement breeding program [35].

##### 2.3.2.2 Influence of genotypes on leaf traits (leaf number per plant, leaf length, width, area, and index) of garlic in Ethiopia

Leaf number is an important trait for obtaining good results in any type of crop production since it is a tool for photosynthesis processes. The food material metabolized in the garlic leaves first flows down to form the garlic bulb. Therefore, the number of garlic leaves per plant is the most important trait that measures garlic plant vigor. However, it was revealed by many researchers' studies that leaf number per plant could be affected by variety, genotype, cultivar, accessions, and germplasms [19,24,27]. According to Ayalew et al. [19], it was indicated that varieties have a significant impact on the number of leaves per plant (p 0.01); a high number of garlic leaves per plant was observed from the local varieties Tseday 92, Kuriftu, and Bishoftu Nech. Contrarily, Mulu et al. [24] reported that a higher leaf number (16.53) was recorded from variety Tsedey 92 than from the local variety, which produced a lower (14.01) number of garlic leaves per plant. According to another study, the variety Bishoftu (standard check) had the most garlic leaves (11) and was statistically similar to germplasm G13 (10.44), G11 (10.22), and G38 (9.89) [27]. The lowest number of garlic leaves (7 and 8) were found in germplasms G48 and G10, respectively [27]. In line with the above report, the maximum number of garlic leaves (6.5 leaves/plant) was observed in the Bisheftu Netch variety, while a small number of garlic leaves (4.75 leaves/plant) was indicated in the Rie local variety [34]. Furthermore, it was revealed that significant (p 0.05) variation in leaf number between the improved garlic varieties is due to genetic variation existing in the different varieties, genotypes, cultivars, accessions, and germplasms. However, some differences revealed between similar varieties indicate different research setups, agro-ecology, and experimental considerations.

Leaf length and diameter are leaf traits that have contributed to the leaf area trait. Scholars revealed that leaf length and diameter were significantly influenced by variety, genotype, cultivar, accessions, and germplasm. The longest leaf (36.51 cm) was observed from the Chelenko I variety, followed by Tsedey (35.44 cm) and local check (35.28 cm), whereas the shortest leaf was recorded from the Chefe variety (31.32 cm) [17]. Similarly, a study conducted in northern Ethiopia [24] revealed that variety Tsedey 92 produced a longer garlic leaf of 44.33 cm and a wider leaf width (2.24 cm), while the local variety produced a shorter garlic leaf length of 35.92 cm and a narrower leaf width (2.02 cm). Similarly, very high, significant variation in leaf length was observed among germplasm collected from northern Ethiopia. The longer (46.11 cm) garlic leaf length was recorded from germplasm G5, which is closely related to germplasm G11 (41.44 cm) and G13 (42.72 cm), whereas the shorter (32.16 and 31.22 cm) garlic leaf length was recorded from germplasm G15 and G48, respectively [27]. According to Abreham et al. [34], among the varieties considered in the experiment, the highest leaf length (30.74 cm) and diameter (1.525 cm) were observed in the improved variety Tsedey 92, while the shortest leaf length (26.07 cm) and diameter (1.224 cm) were found in a local garlic variety. Furthermore, their study did not indicate significant variation (p 0.05) in garlic leaf length between improved garlic varieties. However, in terms of garlic leaf diameter, G-161-2 was found to have the narrowest garlic leaf diameter (1.295 cm) among the considered improved varieties of garlic. Similarly, genotypes G-50/03 and G-7/2003 were recorded as having a shorter (32.3 cm) length, while genotype G-32/2003 was found to have the widest leaf (1.65 cm), and the narrowest (1.35 cm) leaf was recorded for genotype G-21/2003 [31]. Wide leaf area and large leaf area index are large food manufacturing industries that provide large spaces for photosynthetic work, highly affecting garlic yield. A significant variation was revealed among different varieties in leaf area per plant and leaf area index. It shows that Tsedey92 has a high leaf area per plant-1 (6.3% and 7.8%) at 70 and 90 days after emergence, respectively [34]. Besides, higher leaf area and index values (0.71 and 0.94, respectively) were observed for variety Tsedey92 at 70 and 90 DAE, respectively [34]. This is due to the genetic code contributing to large garlic cloves containing more food material reserves, which could have directed the garlic plants to produce larger garlic vigor leaves when compared with small garlic cloves that have comparably smaller food material reserves [27].

##### 2.3.2.3 Influence of genotype on yield and yield traits of garlic in Ethiopia

###### 2.3.2.3.1 Influence of genotype on length and diameter of garlic bulbs in Ethiopia

Yield and yield traits are the most important parameters in any crop. However, these traits were influenced by the genotype (variety) code of genes. Specifically, Mulu et al. [24] revealed that variety Tsedey 92 was recorded as having a long bulb length (4.29 cm), statistically similar to that of the local variety (4.22 cm). The shortest bulb length (3.96 cm) was produced by the variety Bishoftu Netch. This indicates that varieties could vary due to the existence of a genetic information targeted at garlic bulb length. The genotype coded with long bulb length and diameter led to a high garlic bulb yield. Therefore, bulb diameter consideration is of paramount importance. According to Fasikaw et al. [32], it was reported that there is a significant variability among genotypes in bulb diameter (mm). Tsedey 92 (31.2 mm), followed by Chelenko I (27.46 mm), had a significant long bulb diameter, whereas a short garlic bulb diameter (22.98 mm) was observed from a local check [17]. Similarly, a scientific study conducted at northern Ethiopia indicated that the Tsedey 92 variety was recorded as having the longest garlic bulb diameter (4.71 cm), equally with the bulb diameter of 4.62 cm observed from Bishoftu Netch, whereas the shortest bulb diameter (4.36 cm) was obtained from a local variety of garlic [24]. Contrarily, Ayalew et al. [19] reported that local varieties like Kuriftu were scored with a high average bulb diameter compared to varieties like Tseday 92 and MM-98. Contrary reports could be due to genetic differences between local varieties as well as other interacting ecology and agronomic practices. Yebirzaf et al. [27] also collected and characterized different garlic germplasm (Allium sativum L.) for their bulb diameter at Debre Markos, indicating germplasm G18 and G45 had given the highest (3.8 and 3.6 cm) bulb diameter, respectively, while germplasm G48, stand-alone checks Kuriftu and Bishoftu, respectively, had given the lowest bulb diameters of 2.1, 2.16, and 2.22 cm. Surprisingly, the germplasm collected in the area had larger bulb diameters than the improved and released varieties, Kuriftu and Bishoftu.

###### 2.3.2.3.2 Influence of genotype on average garlic bulb weight in Ethiopia

It was reported by different scholars that bulb weight is affected by variety [17,24,34,56]. Azene [56] indicated genetic variability among the varieties in garlic bulb weight. In quantity, a significant high bulb weight (26.18 g) was recorded for variety Tsedey 92, whereas a low bulb weight (17.14 g) was recorded for variety Holeta [17]. As a result of their research, they indicated that using Tsedey 92 instead of local and Chelenko I increased garlic bulb yield by 54.3% and 13.3%, respectively [17]. Similarly, the highest average bulb weight (41.58 g), ranging from 34.31 g to 41.58 g, was shown in variety Tsedey 92, while the lowest (34.31 g) was revealed in a local variety [24]. Furthermore, Abreham et al. [34] observed that Tsedey 92 had the highest mean bulb weight (312.78 g), followed by Bisheftu Netch, while the local variety produced the lowest mean bulb weight (12.33 g). On the other hand, opposite observations were observed from Dabat, Northwestern Ethiopia, indicating the highest fresh bulb weight (49.72 g per plant) indicated by the local variety, followed by Kuriftu (35.36 g per plant), and the lowest bulb weight (16.70 g per plant) observed from Tseday 92. In line with a study reported from Northwestern Ethiopia, Ayalew et al. [19] reported that Tseday 92 had the least bulb dry matter content of 15.33%, while the highest bulb dry matter of 25.83% was observed in the MM-98 variety. Similarly, local Adizemene cultivars had given the highest dry bulb yield (55.44 q/ha), followed by Holleta (42.74 q/ha) and Tsedey (40.88 q/ha) at Angot kebele of Libokmkem District, where Chefe (16.07 q/ha) was produced the lowest yielding dry bulb [26]. According to Tewodros et al. [29], Chelenko I (G-147-2/94) had a superior mean bulb weight of 49.15 g, outperforming the improved variety Tsedey 92 b y 18.18%.

Consequently, the superiority and inferiority of the local variety compared to improved and released varieties under different setups of study might be due to the genetic potential and environmental factors expressing the trait to explore. The variations in garlic bulb weight among varieties could be attributed to vigorous growth, which led to more photosynthetic activity, boosted carbohydrate accumulation, and other physiological processes that resulted in a heavier bulb. Besides, it might be related to their late maturation period, which means they accumulate more food reserves and assimilations. Furthermore, the variability among unregistered germplasms collected from various areas was revealed. The germplasm G18 collected from Awebel-Yazera giorgis was found as the highest bulb weight (21.24 g). However, it was indicated that germplasm G13 from Dejen-Gibgib (17.51) and G5 from Dembecha-Senseb Gebriel (19.90) were not significantly different, whereas the lowest bulb weights (6.63 and 5.34 g) were recorded from germplasm G14 from Dejen-Koncher and G48 from Sekela-Yedem Mariyam, respectively [27].

###### 2.3.2.3.3 Influence of genotype on clove number per bulb and clove length of garlic in Ethiopia

Clove number per bulb and clove length are also the most important garlic yield traits, affected by genotype. According to Atinafu et al. [21], the number of cloves per bulb ranged from 10.80 to 18.95, which was found to be a significant difference between the tested germplasm. The range in their findings indicated the presence of variability among accessions for the number of cloves per bulb hypothesized, and there is significant potential for garlic breeding programs in the future [21]. Accordingly, a study conducted at north Ethiopia indicated that the highest clove number per bulb (23.74) was found in Tsedey 92, whereas the lowest (20.29) was recorded in the local variety [24]. Similarly, the highest average clove length (1.54 cm) was recorded from the improved variety Tsedey 92, while the lowest of 1.17 cm was recorded from the improved variety G-99-2 [34]. Other studies, however, found no significant variation in the number of garlic cloves per bulb among the tested garlic varieties [34]. In contrast to Refs. [24,34], the results reported by Ref. [19] revealed that the variety local had the highest number of garlic cloves per bulb at 20.45, followed by Kuriftu at 16.68, and Tseday 92 had a significantly lower number of garlic cloves per bulb. In addition, Mohammed et al. [17] reported similar findings indicating that the highest number of cloves per bulb was counted from local check (12.75), whereas the lowest was from the variety Holeta (8.36). Interestingly, several researchers indicated that the local variety outperforms the improved and released varieties in the most important yield component trait, which is the edible, medicinally useful, and marketable part of the plant garlic [17,24,34]. Therefore, every researcher should consider local varieties during their studies. In this spirit, other studies confirmed that there was a highly significant difference among the samples collected from different local areas of the country. The high number of garlic cloves per bulb was counted from germplasm G 50, collected from Sekela-Menbeta (21.06), and from G 48, collected from Sekela-Yedem Mariyam (20.40), which was statistically similar to germplasm G10, collected from Bure-Kebsa (18.5), and Kuriftu (17.53), an improved variety [27]. The fewest cloves (10.40, 11.03, and 11.06) were counted from germplasm coded G14, G17, and G15, respectively [27]. Tesfaye et al. [31] also reported that a very high mean number of cloves was recorded for G-34/03 (24.1 cloves per bulb) and a very low number for G-18/03 (7.5 cloves).

###### 2.3.2.3.4 Influence of genotype on marketable and non-marketable cloves and bulb yields of garlic in Ethiopia

The world's production of marketable and non-marketable bulbs is considerable. Therefore, in this section, the review also tried to discuss marketable and non-marketable bulb yields as influenced by the genetic makeup of the garlic planting material. The Tsedey 92 variety produced the highest marketable bulb yield (8.05 t/ha), while a local garlic variety produced the lowest marketable bulb yield (4.944 t/ha) [24]. Variety Tsedey 92 was produced a 38.58% higher marketable bulb yield than the local variety. Besides, Abreham et al. [34] reported that Tsedey 92 was found to be the best variety, which yields the high-marketable garlic clove (311.5 g/plot) and the low-marketable clove (1.3 g/plot) from the local variety and G-99-2. In their study, the Bisheftu Netch variety was also attractive in terms of marketable clove yield in their study. Generally, clove size and morphological features have a direct relationship to total yield, consumer satisfaction, and market need. When and where the marketable garlic clove size and morphological feature increase, garlic bulb yield increases as well, resulting in a fantastic market need, whereas the non-marketable garlic clove has an inverse relationship to yield, consumer satisfaction, and market need, resulting in problems [34].

###### 2.3.2.3.5 Influence of genotype on garlic yield of the total bulb in Ethiopia

In terms of total bulb yield, it was revealed that there is a wide range of variation for bulb yield (from 2003.00 to 7328.00 kg/ha) among germplasm [21]. Specifically, a high yield of 8.45 t/ha was reported from the variety Tsedey 92, whereas a low yield of 4.34 t/ha was reported from the Holeta variety [17]. Similarly, a high total bulb yield (8.98 t/ha) was obtained from variety Tsedey 92; the same was obtained from variety Bishoftu Netch (8.81 t/ha) and the lowest total bulb yield (5.37 t/ha) from a local variety [24]. According to Tewodros et al. [29], Chelenko I (G-147-2/94) had a superior bulb yield (9.33 t/ha). Surprisingly, the bulb yield of the newly developed variety was better than that of the improved Tsedey variety, exceeding it by 14.39%. A trial conducted at south central Ethiopia revealed that the highest bulb yield (80.67 qt/ha) was reported from Tsedey 92, while the lowest (40.32 qt/ha) was produced by the local variety, statistically similar to the G-99-2 variety, at 41.0 qt/ha, and the variety G-161-2 at 43.72 qt/ha [34]. Dessie and Mulat [26] reported that compared with other varieties, Adizemene local and Holleta were recorded as the highest yields in qt/ha at Ginaza kebele. While Tsedey and Kuriftu varieties had low bulb yields of 10.06 q/ha and 11.92 q/ha, respectively, at Woreta, the Chefe variety had a high bulb yield of 22.56 q/ha at Ginaza kebele [26]. Confirming their study, Ayalew et al. [19] also reported the highest yield from the local variety in Northwestern Ethiopia, indicating that varieties released for other areas might not be suitable for different areas. Furthermore, Yebirzaf et al. [27] revealed that germplasm G5 gave the highest yield (7640 kg/ha), which is similar to germplasm G18 (6929 kg/ha), followed by germplasm G38 (4626 kg) and G13 (4601 kg/ha). However, the lowest yield (1471 kg/ha) was harvested from germplasm G48 (1349 kg/ha), Kuriftu (1399 kg/ha), and Bishoftu (1695 kg/ha), respectively [27]. This might be due to the fact that the total bulb yield of garlic is strongly positive and significantly correlated with leaf length, leaf number, clove number, length and width, bulb weight, and clove weight [24].

## 3 Conclusion

Garlic is one of the top medicinal plants due to its high concentration of sulfur and other compounds, which contribute to treating many human diseases. Besides, it is also a vitally important crop for seasoning food around the world. Consequently, garlic has been an economically viable crop worldwide, including in Ethiopia. However, this very important crop, so-called garlic, has been influenced by genotype. This scientific review provides valuable evidence as genotypes influence phenology, growth, yield, and yield-related traits in Ethiopia. The phenology of the garlic crop (days to 50% emergence and days to 75% physiological maturity) is significantly influenced by genotypes. This review indicated that days (time) required for the days to 50% plant emergence ranged from 10 to 15.5 days. In this review, the wide range in 75% physiological maturity (from 94 DAP to 176 DAP) among genotype has been revealed. Growth traits such as plant height was observed in genotypes with a height of 2.32 cm up to 72.83 cm. Whereas genotypes influenced leaf traits such as leaf number per plant, leaf length, diameter, and area index, the ranges were from 4.75 to 16.53, 26.07–46.11 cm, 1.224–2.24 cm, and 0.78 to 0.94, respectively. The most important yield and yield traits are also highly influenced by the genetic code of the planting material and environmental factors for the expression of genes for specific traits. Finally, this review revealed yield and yield traits such as bulb length, diameter, and weight, number of cloves per bulb, clove length, marketable and non-marketable cloves, and total garlic bulb yield, which ranged from very low to high due to genotypes in Ethiopia. Generally, different genotypes influence the phenology, growth, yield, and yield traits of garlic. Therefore, garlic producers should use a high-yielding variety (genotype) in the area. Researchers should conduct additional research into various phonological, growth, yield, and yield traits under various agro-ecologies and agronomic practices. In addition, it is strongly suggested that local varieties be incorporated into any improved and released varieties test.

## Author contribution statement

All authors listed have significantly contributed to the development and the writing of this article.

## Data availability statement

Data included in article/supplementary material/referenced in article.

## Additional information

No additional information is available for this paper.

## Declaration of competing interest

The authors declare that they have no known competing financial interests or personal relationships that could have appeared to influence the work reported in this paper
